# Detailed analysis of the variability of peptidylarginine deiminase type 4 in German patients with rheumatoid arthritis: a case–control study

**DOI:** 10.1186/ar1889

**Published:** 2006-01-16

**Authors:** Berthold Hoppe, Thomas Häupl, Rudolf Gruber, Holger Kiesewetter, Gerd R Burmester, Abdulgabar Salama, Thomas Dörner

**Affiliations:** 1 Institute of Transfusion Medicine, Campus Virchow-Klinikum, Charité-Universitätsmedizin Berlin, Germany; 2 Department of Rheumatology and Clinical Immunology, Campus Charité Mitte, Charité-Universitätsmedizin Berlin, Germany; 3 Out-Patient Clinic for Internal Medicine, Ludwig-Maximilians-Universität München, Germany

## Abstract

Peptidylarginine deiminase type 4 (*PADI4*) genotypes were shown to influence susceptibility to rheumatoid arthritis (RA) in the Japanese population. Such an association could not previously be confirmed in different European populations. In the present study, we analysed exons 2–4 of *PADI4 *in 102 German RA patients and 102 healthy individuals to study the influence of *PADI4 *variability on RA susceptibility by means of haplotype-specific DNA sequencing. Analyses of the influence of *PADI4 *and *HLA-DRB1 *genotypes on disease activity and on levels of anti-cyclic citrullinated peptide antibodies were performed.

Comparing the frequencies of *PADI4 *haplotype 4 (padi4_89*G, padi4_90*T, padi4_92*G, padi4_94*T, padi4_104*C, padi4_95*G, padi4_96*T) (patients, 14.7%; controls, 7.8%; odds ratio = 2.0, 95% confidence interval = 1.1–3.8) and carriers of this haplotype (patients, 27.5%; controls, 13.7%; odds ratio = 2.4, 95% confidence interval = 1.2–4.8), a significant positive association of *PADI4 *haplotype 4 with RA could be demonstrated. Other *PADI4 *haplotypes did not differ significantly between patients and controls. Regarding the individual *PADI4 *variants, padi4_89 (A→G), padi4_90 (C→T), and padi4_94 (C→T) were significantly associated with RA (patients, 49.5%; controls, 38.7%; odds ratio = 1.6, 95% confidence interval = 1.1–2.3). Considering novel *PADI4 *variants located in or near to exons 2, 3, and 4, no quantitative or qualitative differences between RA patients (8.8%) and healthy controls (10.8%) could be demonstrated. While the *PADI4 *genotype did not influence disease activity and the anti-cyclic citrullinated peptide antibody level, the presence of the HLA-DRB1 shared epitope was significantly associated with higher anti-cyclic citrullinated peptide antibody levels (*P *= 0.033).

The results of this small case–control study support the hypothesis that variability of the *PADI4 *gene may influence susceptibility to RA in the German population. Quantitative or qualitative differences in previously undefined *PADI4 *variants between patients and controls could not be demonstrated.

## Introduction

Peptidylarginine deiminases (EC 3.5.3.15) are enzymes involved in the post-translational deimination of protein-bound arginine to citrulline [1]. Five different types of peptidylarginine deiminases encoded by the genes *PADI1*–*PADI4 *and *PADI6 *are currently known [1]. The presence of citrulline-modified target epitopes for autoantibodies is a well-known phenomenon in rheumatoid arthritis (RA) [2,3]. Peptidylarginine deiminases were recently implicated in the generation of anti-cyclic citrullinated peptide antibodies (anti-CCP) detectable in early stages of the disease [2-4]. The process resulting in anti-CCP formation is thought to play a pivotal role in early stages of RA evolvement since it is detectable several years before the onset of symptoms [5]. Certain evidence suggests that deimination of arginine at those peptide side-chain positions that interact with the so-called shared epitope of some major histocompatibility complex class II molecules (for example, HLA-DRB1*0401) may result in the generation of high-affinity peptides, thus inducing a strong *in-vitro *T cell activation [4,6].

A Japanese research group recently identified a genomic region (1p36) containing the genes *PADI1*–*PADI4*, which were suspected to be associated with susceptibility to RA [7]. Peptidylarginine deiminase type 4 (*PADI4*) was identified as the gene actually responsible for the association with RA. *PADI4 *has at least five main haplotypes that differ at four exonic single nucleotide polymorphisms (SNPs) and three subsequent amino acid substitutions [7,8]. While the so-called susceptibility haplotypes 2, 3, and 4 were found to be significantly more frequent in Japanese individuals suffering from RA, the non-susceptibility haplotype 1 predominated in healthy individuals [7]. These results could be confirmed by a further Japanese study [9]. However, studies in different European countries did not reveal significantly different *PADI4 *haplotype distributions in RA patients and healthy individuals. Moreover, no influence of the *PADI4 *genotype on disease severity could be detected [10-14]. Thus, the relevance of *PADI4 *variability for susceptibility to RA is still unclear.

A recent analysis of our group characterising exons 2–4 of the *PADI4 *gene identified new variants and haplotypes by a novel haplotype-specific sequencing-based approach [8]. Importantly, three novel coding SNPs in exons 2, 3, and 4 and three SNPs in introns 2 and 3 located near the exon–intron boundaries were found in 11/102 individuals (10.8%). Moreover, a closely related novel haplotype (haplotype 1B) was found in 2.9% of healthy individuals, which differs from haplotype 1 by padi4_92*G/padi4_96*C [8]. Since this additional variability of the *PADI4 *gene has not been assessed by other studies, the aim of the present case–control study was to investigate the possible influence of *PADI4 *genotypes including previously unknown *PADI4 *variants on susceptibility to RA in a German population.

## Materials and methods

### Subjects and clinical data

Blood samples were obtained from 102 consecutive healthy, unrelated blood donors presenting in our institution as described previously [8]. These samples were analysed in our previous study for genetic variability of exons 2, 3, and 4 of the *PADI4 *gene [8]. Samples from 102 RA patients were enrolled to this study from the Department of Rheumatology, Charité Berlin and from the Rheumatology Unit, Ludwig Maximilian University, Munich. RA patients fulfilled the American College of Rheumatology criteria for RA [15]. The study was approved by the local ethics committee. All individuals were included in this study after informed consent was obtained.

The median age at onset of RA was 47 years (range, 6–86 years). One of the patients (age at onset, six years; *PADI4 *haplotype constellation 1 + 2/3) presented with juvenile RA and later transformed to classical RA. Another patient (age at onset, 14 years; *PADI4 *haplotype constellation 2/3 + 2/3) presented with an early manifestation of classical RA. When excluding these two patients the median age at onset was 48 years (range, 17–86 years). Of the RA patients, 75% were women. The Disease Activity Score 28 was available in 77 cases (median, 5.2; range, 1.8–8.1). Anti-CCP antibodies were detectable in 47 of 75 cases (median, 100 U/ml; range, 0–1600 U/ml). The median age of the controls was 40 years (range, 19–64 years), and 58 (57%) were female.

### Haplotype-specific DNA amplification and DNA sequencing

**Figure 1:**
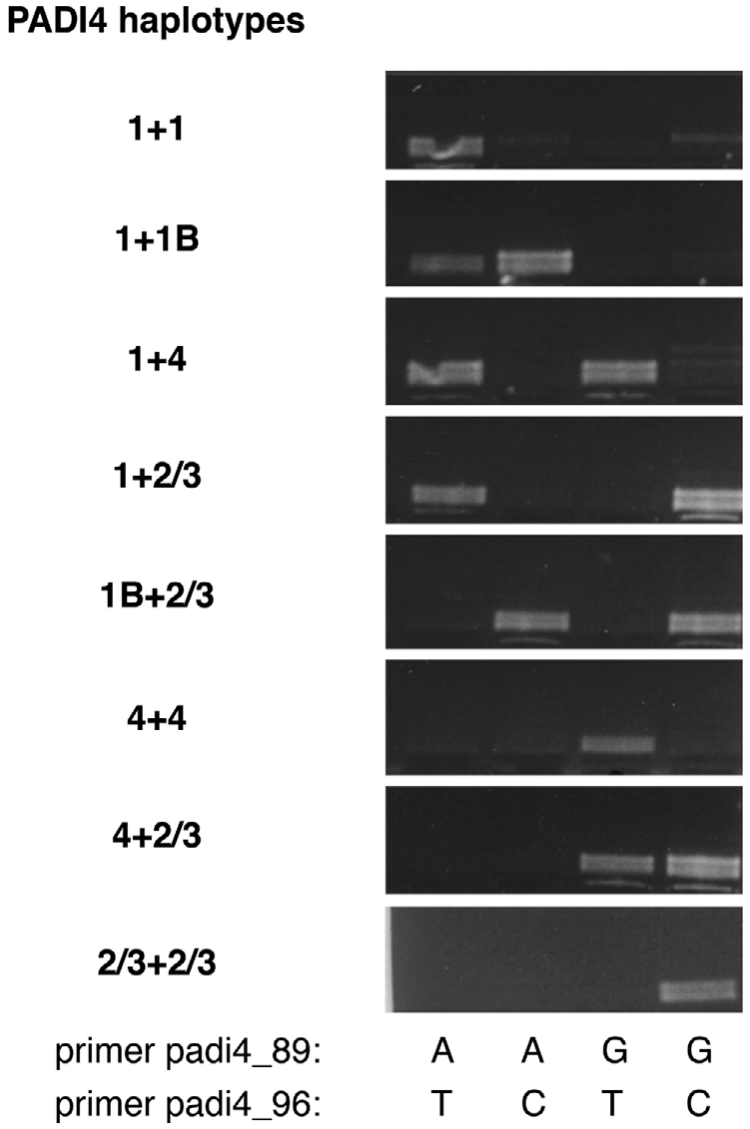
Determination of *PADI4 *haplotype constellations by haplotype-specific long-range PCR. Eight genomic DNA samples with different *PADI4 *haplotype constellations were tested by haplotype-specific long-range PCR using primer mixes specific for padi4_89*A/padi4_96*T (haplotype 1), padi4_89*A/padi4_96*C (haplotype 1B), padi4_89*G/padi4_96*T (haplotype 4), and padi4_89*G/padi4_96*C (haplotype 2/3).

The extraction of genomic DNA, amplification, and cycle sequencing of exons 2–4 of *PADI4 *were performed as described previously [8]. Briefly, the respective *PADI4 *haplotypes were amplified using genomic DNA, primer pairs specific for *PADI4 *haplotype 1, haplotype 1B, haplotype 4, or haplotype 2/3, and Platinum PCR SuperMix High Fidelity (Invitrogen, Karlsruhe, Germany). In most cases the respective *PADI4 *haplotype constellations could be easily identified by gel electrophoretic separation of the amplification products (2% w/v agarose gel containing 0.1 µg/ml ethidium bromide) and UV visualisation (Figure 1).

After digestion of the remaining primers and dNTPs by ExoSAP-IT (Amersham Biosciences, Freiburg, Germany), the PCR products were sequenced. All primers were synthesised by TIB Molbiol (Berlin, Germany). The designations of the *PADI4 *haplotypes are in accordance with those of Suzuki and colleagues [7]. The positions of novel exonic or intronic *PADI4 *variants were designated relative to sequences NM_012387 and NT_034376.1, respectively.

### HLA-DRB1 genotyping, definition of the shared epitope, and anti-CCP measurement

Sequencing-based high-resolution typing of HLA-DRB1 was performed in 58 cases using the Protrans S4 HLA-DRB1 kit (lot number 344A01; Protrans, Ketsch, Germany) as previously described [16]. Presence of the shared epitope was assessed in two ways. First, only HLA-DRB1*0401, HLA-DRB1*0404, and HLA-DRB1*0408 were considered. Second, the shared epitope was defined by all HLA-DRB1 alleles with the following constellations: DRß1 (67Leu–69Glu–71Lys or Arg–74Ala–86Gly or Val) [17]. Anti-CCP antibodies were measured in 75 cases using standard techniques [18].

### Statistical analysis

Chi-square tests (odds ratio, 95% confidence interval) and Fisher's exact tests were performed using GraphPad Prism 4 (GraphPad Software, San Diego, CA, USA). Comparison of the serum anti-CCP levels and the Disease Activity Score 28 regarding dependence of the *PADI4 *and *HLA-DRB1 *genotypes was assessed by the Mann–Whitney U test (median and 25th–75th percentiles are presented).

Chi-square testing for deviation from Hardy–Weinberg equilibrium was performed by a Java-based applet (Knud Christensen, Department of Animal and Veterinary Basic Sciences, Denmark; ).

## Results

### Distribution of *PADI4 *haplotype combinations

The frequencies of the *PADI4 *haplotype combinations found in our study are presented in Table 1. A detailed description of the variability of exons 2–4 of the *PADI4 *gene in healthy individuals analysed by haplotype-specific DNA sequencing was given in our previous report [8]. *PADI4 *haplotype 1 was most frequently found in the homozygous form (34.3%) and in combination with haplotype 2/3 (34.3%) in normal controls. In contrast, *PADI4 *haplotype 1 occurred more frequently in combination with haplotype 2/3 (30.4%) than in the homozygous form (24.5%) in patients with RA. Most strikingly, the frequency of combined *PADI4 *haplotype 1/haplotype 4 was significantly different between patients (19.6%) and controls (8.8%) (*P *< 0.05). Both in patients and controls the distributions of the *PADI4 *haplotype combinations were in accordance with Hardy–Weinberg equilibrium.

### Frequencies of *PADI4 *haplotypes and carriers of *PADI4 *haplotypes

When we compared the overall frequency of haplotype occurrence, haplotype 4 of *PADI4 *was significantly more prevalent in RA patients (14.7%) than in controls (7.8%) (odds ratio = 2.0, 95% confidence interval = 1.1–3.8, *P *= 0.04) (Table 2). The frequency of carriers of *PADI4 *haplotype 4 also differed significantly between patients (27.5%) and controls (13.7%) (odds ratio = 2.4, 95% confidence interval = 1.2–4.8, *P *= 0.02). For all other *PADI4 *haplotypes, there were no significant differences between patients and controls.

### Frequencies of *PADI4 *SNPs and novel *PADI4 *variants

The haplotype-specific sequencing based approach used in this study covered the genomic regions of exons 2, 3, and 4 of *PADI4 *and included the SNPs padi4_89, padi4_90, padi4_92, padi4_94, padi4_104, padi4_95, and padi4_96. The approach used therefore allowed a very detailed analysis of this part of the *PADI4 *gene that was implicated in influencing RA susceptibility. Of these SNPs, the frequencies of padi4_89A→G, padi4_90C→T, and padi4_94C→T in the RA patients (49.5%) were significantly different from those in the controls (38.7%) (Table 3). The resulting odds ratio was 1.6 (95% confidence interval = 1.1–2.3, *P *= 0.04).

In an earlier study [8], six previously unknown *PADI4 *variants were discovered in 11 (10.8%) of the healthy controls included in the present study. Three of these resulted in amino acid substitutions. Nine (8.8%) of the RA patients from the present study exhibited five of these new *PADI4 *variants – 265G→A (D89T) (*n *= 2), 390194C→T (*n *= 1), 304C→A (P102T) (*n *= 1), 393030A→G (*n *= 1), and 392G→C (R131T) (*n *= 3) – and another previously unknown *PADI4 *variant – 236C→G (T79R), EMBL AJ966355 (*n *= 1). Comparison of these *PADI4 *variants did not reveal any significant quantitative or qualitative differences between patients and controls.

### Influence of *PADI4 *genotype on anti-CCP level and disease activity

When comparing anti-CCP levels in carriers versus non-carriers of *PADI4 *haplotype 1 (median, 100 [0–437] U/ml versus 102 [0–644] U/ml; *P *= 0.69), haplotype 2/3 (median, 183 [0–651] U/ml versus 73 [0–200] U/ml; *P *= 0.13), and haplotype 4 (median, 71 [0–200] U/ml versus 183 [0–620] U/ml; *P *= 0.15), no significant influence of *PADI4 *genotype on anti-CCP level could be detected. Anti-CCP levels in *PADI4 *haplotype 1, haplotype 2/3, and haplotype 4 homozygotes were also not different. The disease activity measured by Disease Activity Score 28 differed non-significantly in carriers versus non-carriers of *PADI4 *haplotype 1 (median, 5.3 [4.3–6.3] versus 4.8 [3.5–5.7]; *P *= 0.17), haplotype 2/3 (median, 5.0 [3.9–5.9] versus 5.5 [4.6–6.4]; *P *= 0.23), and haplotype 4 (median, 5.2 [3.9–6.6] versus 5.2 [4.1–5.9]; *P *= 0.73).

### Influence of *HLA-DRB1 *genotype on anti-CCP level

The presence of the shared epitope, defined by the HLA-DRB1 alleles HLA-DRB1*0401, HLA-DRB1*0404, and HLA-DRB1*0408 (shared epitope present; median, 607 [17–1170] U/ml versus 0 [0–392] U/ml; *P *= 0.048) or by DRβ1 (67Leu–69Glu–71Lys or Arg–74Ala–86Gly or Val; median, 607 [0–1170] U/ml versus 0 [0–252] U/ml; *P *= 0.033), significantly influenced the level of anti-CCP.

## Discussion

This study provides a hint that variability of the *PADI4 *gene is related to the susceptibility to RA in the German population, whereas certain differences of hitherto unknown *PADI4 *variants between patients and controls were not found. The impact of *PADI4 *genotypes on susceptibility to RA remains controversial [7,9-14]. Until now, certain *PADI4 *genotypes (haplotypes 2, 3, and 4) have been implicated to be involved in the pathogenesis of RA only in Japanese populations [7,9]. No such association of *PADI4 *variability with RA prevalence and severity could be demonstrated in various European populations [10-14]. In our study, also, an influence of *PADI4 *genotype on disease activity or anti-CCP level could not be demonstrated. The mechanism by which *PADI4 *variability may influence the break of tolerance is still unknown. Initially, it was argued that detectable differences in mRNA stability could result in higher enzymatic activity in cases where the susceptibility haplotypes (2,3 and 4) of *PADI4 *are present, leading to the generation of larger amounts of citrullinated peptides [7]. Most recently, a close association of the production of anti-CCP antibodies and HLA-DRB1 has been described [6,11,13,19], indicating the importance of antigen presentation in the induction of autoimmunity. This finding clearly could be confirmed in our study.

With the exception of haplotype 4, the frequencies of all other *PADI4 *haplotypes in our control individuals were comparable with those reported by other groups [7,9,10,14]. While the frequency of *PADI4 *haplotype 4 in our study (7.8%) was similar to that reported by groups from the United Kingdom (9.4%, *P *= 0.51; here termed *haplotype 3*) [10], Spain (5.9%, *P *= 0.32; padi4_94*T, padi4_104*C) [14], and Japan (5.5%, *P *= 0.17) [9], it was statistically significant different from the frequency reported by the large initial Japanese study (4.0%, *P *= 0.013) [7]. All of our patients and healthy individuals were Caucasian. The fact that the *PADI4 *haplotype 4 frequency in our control population was significantly higher compared with one of the Japanese studies [7] may therefore be influenced by differences in the ethnic background.

In our study, a statistically significant positive association of *PADI4 *haplotype 4 with RA was observed (odds ratio = 2.0, 95% confidence interval = 1.1–3.8). The presence of this haplotype did not influence disease activity or the anti-CCP level. We did not found an association of RA and *PADI4 *haplotypes 2 and 3, which were described as the principal susceptibility haplotypes in the Japanese population [7]. However, we cannot exclude that this difference may be influenced by the size of our study population.

When analysing the distributions of those *PADI4 *SNPs covered by our genotyping approach, padi4_89A→G, padi4_90C→T, and padi4_94C→T were found to be significantly associated with RA. These SNPs are common with *PADI4 *haplotype 4 and haplotype 2/3, whereas padi4_104C→T, padi4_95G→C, and padi4_96T→C, which are common with *PADI4 *haplotype 4 and haplotype 1, exhibited no association with RA.

The present study identified uncommon *PADI4 *variants that are not typically included among the five main *PADI4 *haplotypes. Consistent with our previous findings in healthy individuals [8], this study also revealed additional variability in *PADI4 *exons 2–4 in RA patients. As a result of this study, the frequency of uncommon *PADI4 *variants as identified earlier [8] was apparently not different quantitatively or qualitatively between patients and controls.

Of note, a statistically significant association between certain *PADI4 *genotypes and RA was detected in our study, in contrast to reports from other European groups [10-14]. This puzzling discrepancy may be due to influencing factors, such as a homogeneous Caucasian population, although we cannot definitely exclude other selection biases.

The question of whether PADI4 variability alters the interactions between the enzyme and possible target proteins remains unclear [20]. Further studies are needed to characterise the influence of this variability on the repertoire of deiminated target proteins.

## Conclusion

In summary, the *PADI4 *haplotype 4 and the SNPs padi4_89A→G, padi4_90C→T, and padi4_94C→T were found to be significantly associated with RA in a German population. The genomic region of *PADI4 *exons 2–4 of RA patients exhibits additional variability, which is apparently not different quantitatively and qualitatively between RA patients and controls. While the *PADI4 *genotype did not influence disease activity or the anti-CCP level, the presence of the HLA-DRB1 shared epitope was associated with significantly higher anti-CCP levels.

## Abbreviations

anti-CCP = anti-cyclic citrullinated peptide antibodies; PADI4 = peptidylarginine deiminase type 4; PCR = polymerase chain reaction; RA = rheumatoid arthritis; SNP = single nucleotide polymorphism.

## Competing interests

The authors declare that they have no competing interests.

## Authors' contributions

BH participated in the design and coordination of the study, carried out the molecular genetic and statistical analyses, and drafted the manuscript. TH, RG, HK, GRB, and AS participated in the coordination of the study and in drafting the manuscript. TD participated in the design and coordination of the study, and critically revised the manuscript. All authors read and approved the final manuscript.

## Figures and Tables

**Table 1:** *PADI4 *haplotype combinations in Caucasian individuals

Haplotype A	Haplotype B			
	
	Haplotype 1	Haplotype 1B	Haplotype 2/3	Haplotype 4
Haplotype 1				
Controls	35 (34.3%)	5 (4.9%)	35 (34.3%)	9 (8.8%)*
Patients	25 (24.5%)	1 (1%)	31 (30.4%)	20 (19.6%)*
Haplotype 1B				
Controls	0	0	1 (1%)	0
Patients	0	0	0	0
Haplotype 2/3				
Controls	0	0	12 (11.8%)	3 (2.9%)
Patients	0	0	17 (16.7%)	6 (5.9%)
Haplotype 4				
Controls	0	0	0	2 (2%)
Patients	0	0	0	2 (2%)

Frequencies of different *PADI4 *haplotype combinations in patients with rheumatoid arthritis (*n *= 102) and in healthy controls (*n *= 102) are presented. *P < 0.05 (Fisher's exact test).

**Table 2:** *PADI4 *haplotype frequencies in Caucasian individuals

	*PADI4 *haplotype (frequencies)				*PADI4 *haplotype (carriers)			
	
	Haplotype 1	Haplotype 1B	Haplotype 2/3	Haplotype 4	Haplotype 1	Haplotype 1B	Haplotype 2/3	Haplotype 4
Controls	119 (58.3%)	6 (2.9%)	63 (30.9%)	16 (7.8%)	84 (82.4%)	6 (5.9%)	51 (50%)	14 (13.7%)
Patients	102 (50%)	1 (0.5%)	71 (34.8%)	30 (14.7%)	77 (75.5%)	1 (1%)	54 (52.9%)	28 (27.5%)
Odds ratio	0.71	0.16	1.2	2.0	0.66	0.16	1.1	2.4
95% confidence interval	0.48–1.1	0.02–1.4	0.79–1.8	1.1–3.8	0.33–1.3	0.02–1.3	0.65–1.9	1.2–4.8
*P*	0.11	0.12	0.46	0.04	0.30	0.12	0.78	0.02

*PADI4 *haplotype and carrier frequencies in patients with rheumatoid arthritis (*n *= 102) and in healthy controls (*n *= 102) are presented. Results of univariate analyses (odds ratio, 95% confidence interval) and Fisher's exact tests are indicated.

**Table 3:** Frequencies of *PADI4 *variants in Caucasian individuals

	padi4_89 (A→G)	padi4_90 (C→T)	padi4_92 (C→G)	padi4_94 (C→T)	padi4_104 (C→T)	padi4_95 (G→C)	padi4_96 (T→C)
Controls	79 (38.7%)	79 (38.7%)	97 (47.5%)	79 (38.7%)	63 (30.9%)	63 (30.9%)	69 (33.8%)
Patients	101 (49.5%)	101 (49.5%)	102 (50%)	101 (49.5%)	71 (34.8%)	71 (34.8%)	72 (35.3%)
Odds ratio (95% confidence interval)	1.6 (1.1–2.3)	1.6 (1.1–2.3)	1.1 (0.8–1.6)	1.6 (1.1–2.3)	1.2 (0.8–1.8)	1.2 (0.8–1.8)	1.1 (0.7–1.6)
*P*	0.04	0.04	0.69	0.04	0.46	0.46	0.84

The allele frequencies of *PADI4 *variants in patients with rheumatoid arthritis (*n *= 102) and in healthy controls (*n *= 102) are presented. Results of univariate analyses (odds ratio [95% confidence interval]) and Fisher's exact tests are indicated.
